# LC–MS/MS quantification of bacterial and fungal signal peptides via direct injection: a case study of cross-kingdom communication

**DOI:** 10.1007/s00216-025-05767-6

**Published:** 2025-02-04

**Authors:** Carolin Pohl, Linda Schuster, Cindy Rau, Uta Gutbier, Stephan Beil, Hilmar Börnick, Kai Ostermann, Stefan Stolte

**Affiliations:** 1 Faculty of Environmental Science, Institute of Water Chemistry, TUD Dresden University of Technology, 01062 Dresden, Germany; 2 Faculty of Civil Engineering, Division of Water Science, HTW University of Applied Sciences, Friedrich-List-Platz 1, 01069 Dresden, Germany; 3 Else Kröner Fresenius Center for Digital Health, Faculty of Medicine Carl Gustav Carus, TUD Dresden University of Technology, Dresden, Germany; 4 Faculty of Biology, Research Group Biological Sensor-Actuator-Systems, TUD Dresden University of Technology, 01062 Dresden, Germany

**Keywords:** Cross-kingdom communication, LC–MS/MS, Signal peptide, Yeast

## Abstract

Bacteria and yeast use secreted signal peptides, also known as pheromones, for cell–cell communication within their respective species. Recently, genetic modification has allowed for the extension and exploitation of this type of communication, to communication between organisms from different species and even from different kingdoms. This innovative approach is intended to allow for the large-scale production of specific compounds for applications in medicine and biotechnology while producing reduced amounts of by-products. Until now, the detection of signal peptides, which are often short-lived and only present in small amounts, is usually qualitative, non-selective, and time-consuming and/or requires the presence of additional cell types. Here, an ESI-LC–MS/MS method for the specific quantification of signal peptides from yeast (α- and P-factor) and bacteria (CSF) using a TSKgel column operating under HILIC conditions has been demonstrated. The influence of different matrices, their adsorption behavior, and their stability were investigated. In matrix, LOQs of 0.05 µM, 0.03 µM, and 0.02 µM were obtained for CSF, α-factor, and P-factor, respectively. Subsequently, the developed method was applied to the detection of yeast- and bacteria-specific peptides secreted by genetically modified yeasts. It could be demonstrated that under overexpressing conditions, α-factor and P-factor concentrations of 1 µM were measured, while for CSF concentrations as high as 2.5 µM was reached. Finally, the established method permits the simultaneous, quantitative detection of signal peptides in different matrices and without pre-concentration in near-real time, thus advancing the possibility of tracking cross-kingdom communication.

**Graphical Abstract:**

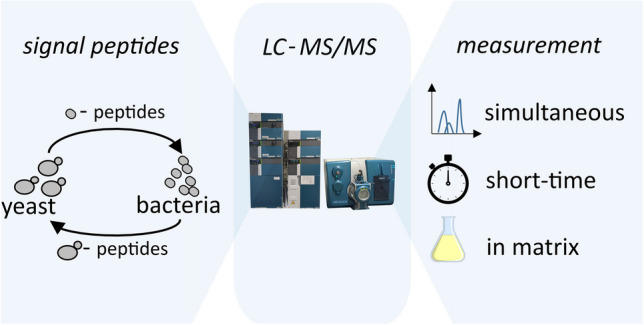

**Supplementary Information:**

The online version contains supplementary material available at 10.1007/s00216-025-05767-6.

## Introduction

Cell–cell communication between individuals within a species is a naturally occurring process and is of great importance to both eukaryotes and prokaryotes [1]. For instance, the yeast *Saccharomyces* (*S.*) *cerevisiae* releases pheromones to initiate the pheromone response pathway of *a* and *α* haploid cells in order to form diploid cells [2, 3]. Meanwhile, the bacterium *Bacillus* (*B.*) *subtilis* secretes an extracellular peptide, competition and sporulation factor (CSF), to form endospores once a certain population density is achieved—a process which referred to as quorum sensing [4]. The fundamental principle of communication is consistent: a signal is produced by a cell (= sender cell) and released into the surrounding environment where it encounters another cell (= receiver cell), binds to the cell’s receptor, and activates a response pathway [5]. The activation of such pathway results in coordinated behavior allowing for adaptation to environmental changes. Such coordinated behavior includes changes in cellular metabolism and homeostasis and regulation of growth and development [6]. Often, the communication is based on biochemical signaling molecules including small molecules (e.g., acetaldehydes), peptides (e.g., pheromones), lipids (e.g., oxylipins), and biomacromolecules [5–7].

For biotechnological purposes, artificial cell–cell communication pathways have been developed for single populations or mixed communities of species to achieve controlled responses. For example, to support the production of organic compounds with higher yields and with fewer cross-reactions and by-products ( [8–10]). Shin et al. engineered two different *Escherichia coli* strains for the fermentation of xylan to ethanol [9]. Meanwhile, Tsai et al. used four different engineered yeast strains to degrade cellulose to ethanol [10]. In the field of medical technology, Hwang et al. engineered *Escherichia coli* to specifically recognize, migrate toward, and eradicate *Pseudomonas aeruginosa* [11]. Besides this, Williams et al. used one yeast strain to produce a pheromone (α-factor) and a second strain which receives the α-factor and produces GFP (green fluorescent protein) in response [12].

A novel approach represents the cross-kingdom communication between prokaryotes and eukaryotes, as initially reported by de Luis et al. in 2022 [13]. Typically, prokaryotes communicate via quorum sensing molecules and yeast via mating factors, two different structures and a reason why cross-kingdom communication rarely occurs naturally [13]. The heterogeneous composition of a mixed cross-kingdom community offers the potential to combine the strengths and properties of both kingdoms (e.g., bacteria and yeast) along with the possibility of integrating a greater number of genes and functions to enable a certain task.

A typical signal peptide from *S. cerevisiae* is the α-factor, which causes cell cycle arrest in the opposite *a*-yeast cell type by interacting with a receptor (for a detailed description, see [14]). This in turn results in a proteolytic degradation of α-factor by the Bar1p protease [15]. In the bacteria *B. subtilis*, the extracellular peptide CSF plays an important role in communication, controlling competence, and sporulation [16] and is taken up by the cell via a permease [4, 16]. In both organisms, the signal peptide itself is short-lived, as it is rapidly degraded in order to terminate the message. Additionally, such signal peptides are usually present at low concentrations [5]. To initiate cross-kingdom communication, organisms must be engineered to respond to signaling molecules from another kingdom. For example, Vološen et al. used a genetically engineered strain of *S. cerevisiae* to secrete the bacteria-specific peptide CSF into the surrounding culture media during mixed cultivation. An also present engineered strain of *B. subtilis* than responded to the yeast-derived CSF with the production of bioluminescence [17].

In the context of cross-species and cross-kingdom communication, an analytical method is required to quantify mixtures of signal peptides in relevant concentration ranges for verification of a response. This will enable the study of production and availability in sender and receiver cells during cross-kingdom communication.

To date, the measurement of CSF has been carried out via β-galactosidase activity [16, 18–20] or luciferase activity [17] while α-factor has been detected via the observation of morphological changes (Shmoo effect), the halo assay, through β-galactosidase activity [14, 21] or via ELISA [22]. However, none of these methods is capable of the rapid, in-matrix, high-throughput, and simultaneous measurement of multiple signal peptides. For instance, the ELISA method is sensitive (LOD 0.1 nM); however, it is also very time-consuming (long incubation time > 24 h) and requires a significant amount of manual labor (multiple pipetting steps) [22]. Furthermore, the specificity of a given antibody for a single peptide, combined with the limited availability of antibodies for most signal peptides, does not allow for simultaneous quantification. In addition, it is not possible to measure at low pH values, as the antibodies can denature [22]. Consequently, to optimize cross-species and cross-kingdom communication, there is a need for an assay that allows for the rapid, simultaneous quantification of multiple peptides in culture medium.

This study presents a rapid, robust, and sensitive analytical method for the simultaneous quantification of bacterial and fungal signal peptides from complex matrices without time-consuming and error-prone pre-concentration steps to follow cross-kingdom communication. The method uses hydrophilic interaction liquid chromatography (HILIC) coupled to ESI–MS/MS. For the case study, genetically modified yeast species (*S. cerevisiae* and *Schizosaccharomyces* (*S.*) *pombe*) are used. These yeast strains are able to overexpress and secrete their own pheromones (α- and P-factor) as well as a bacterial-specific peptide (CSF). The selected peptides differ in structure and polarity, as illustrated in Table 1. Yeast α-factor “WHWLQLKPQPMY” is positively charged with hydrophobic side chains or aromatic ring systems. In contrast, the CSF peptide “ERGMT” is uncharged and contains rather polar amino acids, while the P-factor “TYDAFLRAYQSWNTFVNPDRPNL” has aromatic rings and some polar side chains. Once the method has been established and validated, it will be possible to analyze secreted peptides almost in real time in the culture supernatant, and thus advance the development of cross-kingdom communication.

**Table 1:**
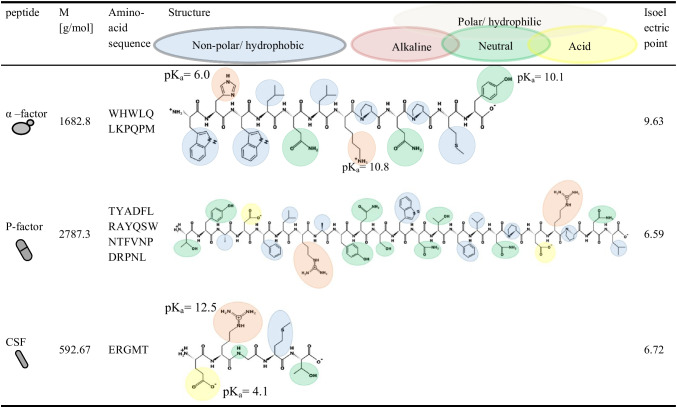
Structural overview of the peptides used in this study including molar mass (M), amino acid sequence, structure with pK_a_ (acid dissociation constant), and the isoelectric point (structure and calculation using https://pepdraw.com/)

## Material and methods

### Chemicals and materials

Acetonitrile (ACN, 98% purity), pure water (HPLC grade), acetic acid LiChropur® for LC–MS (AA, 100% purity), formic acid HiPerSolv CHROMANOM® for LC–MS/MS (FA, ≥ 99% purity), and ammonium formate (NF, ≥ 99% purity) were purchased from VWR International GmbH (Germany). Ammonium acetate (NA, ≥ 96% purity) was ordered from Carl Roth (Germany). The *S. cerevisiae* α-mating factor was ordered from UMEX GmbH (Germany) and the *B. subtilis* CSF signal peptide was custom synthesized by peptides&elephants GmbH (Germany). The P-pheromone from *S. pombe* was obtained from Davids Biotechnologie (Germany). All stock solutions were stored at − 20 °C as aliquots with a final concentration of 5 mM. Working solutions of 100 µM were prepared in HPLC-grade water with 0.1% AA and stored at 4 °C. All internal standards (ISTDs) were custom synthesized by PSL GmbH (Germany) and stored in HPLC-grade water with 0.1% FA at − 20 °C in aliquots. The α-factor ISTD (WHWL(^13^C_6_,^15^N_2_)QL(^13^C_6_,^15^N_2_)KPQPMY, 1698.8 g/mol) and the P-factor ISTD (TYADFL(^13^C_6_,^15^N)RAYQSWNTF(^13^C_9_,^15^N)VNPDRPNL, 2804.3 g/mol) were stored at a concentration of 200 µM and the CSF-ISTD (ER(^13^C_6_, ^15^N_4_)GMT, 603.3 g/mol) at a concentration of 500 µM. For analysis, solutions containing 5 μM α-factor-ISTD, 10 μM CSF-ISTD, and 5 μM P-factor-ISTD in 500 mL HPLC-grade water with 0.25 mL FA and 500 mL ACN were prepared.

The yeast strain *S. cerevisiae* BY4717Δ*bar1* was genetically modified by transformation with p425 derivates as described by Vološen et al. [17]. The plasmid variants harbor different combinations of up to four open reading frames for the different signal peptides (Table 2). Cultivation of *S. cerevisiae* was done in W0 medium (1.7 g/L yeast nitrogen base, 5 g/L ammonium sulfate, 20 g/L glucose, pH 5.6) supplemented with 60 mg/L L-histidine, 80 mg/L L-leucine, and 20 mg/L L-methionine. For *S. pombe*, the different constructs encoding for the signal peptides (Table 2) were synthesized similarly to the procedure in *S. cerevisiae*. The constructs were integrated into vector pJR1-3XL [23] and transformed in the *S. pombe* strain HE620 [24] by the lithium acetate method described by [25]. For the cultivation of *S. pombe* HE620, Edinburgh minimal medium (EMM, pH 6) was used, as described in [26]. Furthermore, a mineral medium (MV medium, pH 6) according to [17] was prepared. This MV medium supports similar growth characteristics for yeast (*S. cerevisiae*) and bacteria (*B. subtilis*) [27]. The plasmids transformed into the modified yeasts used in this study are presented in Table 2.

**Table 2:** Details of the peptide encoding genes carried on specific plasmids and expressed in specific yeast strains

Plasmid	Vector backbone	Construct	Encoded signal peptides			
*S. cerevisiae* BY4717 Δ*bar1*	p425 GPD	Control	Empty vector			
		2α2CSF	α	α	CSF	CSF
		1CSF			CSF	
		4CSF	CSF	CSF	CSF	CSF
*S. pombe* HE620	pHR1-3XL	Control	Empty vector			
		2P2CSF	P	P	CSF	CSF
		4CSF	CSF	CSF	CSF	CSF
		1P1α1CSF	P	α	CSF	CSF

### Development of the HPLC–MS/MS multimethod

For eluent optimization, a Series 1100 HPLC (Agilent, USA) coupled to a tandem mass spectrometer QTRAP®3200 (Sciex) with an integrated ESI interface was used. Subsequent experiments, including gradient optimization, validation, and peptide analysis, were conducted using a UHPLC system (Shimadzu Nexera X2 LC-30AD) coupled to a tandem mass spectrometer (QTRAP®6500 + , Sciex). In both cases, the data evaluation was performed using the MultiQuantTM 3.0.3 software.

#### LC–MS parameter

The selected MRM transitions and the MS parameters are presented in the SI (Table S1). For LC separation, a TSKgel Amide-80 3 µm 2.0 × 150 mm (TOSOH Bioscience) column was used in HILIC mode for peptide analysis. Based on an isocratic elution (75% eluent A— HPLC-grade water with 0.1% AA, 25% eluent B—ACN with 0.1% AA), the peak shapes and retention times were optimized by varying the type and amount of acid and buffer according to Table 3 over an analysis time of 25 min.

**Table 3:** Variation of acid type and concentration as well as buffer type for optimization of the eluent composition

Acid	Acid concentration (%)	Salt/buffer
Acetic acid	0.01	
	0.04	
	0.1	10 mM ammonium acetate
Formic acid	0.125	10 mM ammonium formate

Furthermore, a gradient was developed in order to achieve an appropriate peptide separation and to enable matrix quantification. Peptides were applied to the column using a constant initial eluent composition that allowed for complete binding of the peptides to the column while eluting major matrix components. The initial eluent composition was kept constant for 2, 7, 12, 17, and 22 min (= focusing time), to achieve optimal separation from matrix components before starting the peptide elution. For this purpose, 100 µL of a 10µM sample (CSF, α-factor) was mixed with 900 µL of fresh W0 or MV media. As a reference, the peptides were diluted in matrix-free HPLC-grade water containing 0.125% FA.

#### Investigation of peptide stability in matrices and on surfaces

##### Sorption to different vessel surfaces depending on matrix compounds

To evaluate the sorption of peptides to surfaces and the effect of different matrices, the influence of vial inserts made of glass, silanized glass, and polypropylene was tested by adding 150 µL of 1 µM synthetic peptide mix into each insert. The peptides were dissolved in different matrices: HPLC-grade water with 0.125% FA; fresh MV media; and HPLC-grade water with 30% ACN and 0.125% FA. The remaining peptide concentration was analyzed at 3h intervals over 22 h in total.

##### Stability in the yeast cultivation supernatant

Stability of the peptides was analyzed in freshly prepared media and in the supernatant from yeast cultivations in W0 and MV media. For this purpose, the yeast cultivations were initiated in 10 mL media with an optical density (OD_600nm_) of 1 and were cultivated for 24 h at 30 °C with continuous shaking. Subsequently, 2 mL of the culture was sampled and centrifuged at 5000 × g for 5 min. The resulting supernatant was removed and stored at -20 °C until LC–MS analysis. To study the time-dependent peptide stability, yeast supernatant and fresh culture medium with a volume of 900 µL each were spiked with 100 µL of a synthetic peptide mixture containing 10 µM of each α-factor and CSF in HPLC-grade water with 0.125% FA. The samples were analyzed immediately after sampling and subsequently over seven 3h intervals, in order to determine the remaining peptide concentrations. Furthermore, 6 M urea, 30% ACN and heating of the sample for 3 min to 80 °C, was tested for their suitability as conservation procedures. The results were compared to those obtained for peptides merely dissolved in HPLC-grade water with 0.125% FA.

#### Validation

A validation of the peptide quantification method developed concerning linearity, LOD and LOQ, precision, selectivity, and robustness was conducted in a mixture consisting of 50% ACN and 50% supernatant from the yeast strain BY4741Δ*bar1*–p425 GPD in MV medium following 24 h of cultivation. Calibration samples were prepared in the range of 0.01 to 1 µM using a synthetic peptide mix of α- and P-factor and CSF. To 150 µL samples, 10 µL of the ISTD-mix (containing 5 µM α-factor, 5 µM P-factor, and 10 µM CSF) was added. LOD and LOQ were determined using the calibration line method according to DIN32645 (10-point calibration). The intra- and inter-day precision were calculated by measuring the peptide mix (α- and P-factor, CSF) at three concentration levels (0.05, 0.2, and 0.7 µM) six times on 1 day, and by repeating this measurement on three further, separate days. Selectivity was determined by obtaining the background noise at the retention times of the peptides using five blank samples consisting of 50% ACN and 50% cell supernatant after 24 h of cultivation. The robustness of the method was analyzed by double determinations of α- and P-factor and CSF in the concentrations 0.05, 0.2, and 0.7 µM in different matrices, including W0 and MV media (fresh and in the supernatant) as well as HPLC-grade water with 0.125% FA. The validation results are presented in the SI.

### Case study: analysis of peptides secreted from yeast

For the measurement of secreted peptides, the corresponding genetically modified yeast strain (see Table 2) was cultivated in culture medium (V = 20 mL) and 2 mL samples were removed at 0, 2, 4, 6, and 24 h of cultivation. The samples were centrifuged at 5000 × g for 5 min; the supernatant was sampled and frozen at -20 °C. For analysis, 75 µL of each sample was mixed with 75 µL ACN and spiked with 10 µL of the ISTD.

## Results and discussion

### LC–MS/MS method development

Initially, the method was developed for two distinct peptides: α-factor and CFS. Subsequently, P-factor was incorporated and the method was finalized. The subsequent validation was carried out for all three signal peptides simultaneously.

#### Development of a suitable HPLC separation method

Reliable quantification of signal peptides requires an appropriate retention time and peak shape. In simple terms, the separation of peptides in HILIC is based on interactions between the hydrophilic stationary phase and the rather hydrophobic organic mobile phase. By applying isocratic conditions (Fig. S1) in a solution consisting of 75% HPLC-grade water and 25% ACN, the influence of acid type (AA, FA), acid content (0.01–0.125%), and the presence of salts (ammonium formate and ammonium acetate) was investigated (Fig. S2). The lower pH of the eluent results in an increase in the protonation of α-factor and CSF, which consequently reduces their interaction with the carbamoyl group of the stationary phase. This leads to a shorter retention time and smaller peak width. The addition of buffer resulted in narrower peaks for α-factor and CSF through reduced electrostatic interactions. The optimized eluents consist of eluent A: 950 mL HPLC-grade water, 50 mL ACN, 1.25 mL FA, 10 mM ammonium formate, and eluent B: 950 mL ACN, 50 mL HPLC-grade water, and 1.25 mL FA.

**Fig. 1:**
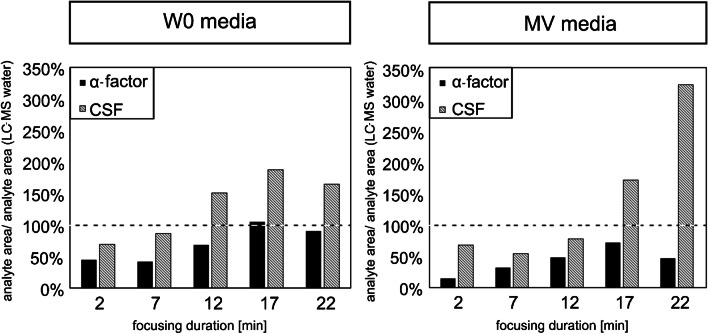
The influence of the matrix (W0 and MV media) on the peptide peak area as a function of increasing focusing time (2, 7, 12, 17, and 22 min) is shown. The analyte (peptide) peak area in matrix is reported as a percentage relative to the peak area of the peptide run in HPLC-grade water. The 100% line symbolizes equivalent peak area ratio in the HPLC-grade water samples

Furthermore, a gradient has been developed by systematic variation in order to separate the overlapping peaks and to avoid co-elution with the dead volume at 1.1 min and interferences with matrix components. Matrix components can lead to ion suppression or enhancement by co-elution and binding or competition for charges with the analyte. Various techniques have been developed to address these limitations, including solid-phase extraction (SPE), protein precipitation (PP), and liquid–liquid extraction (LLE). However, these methods often result in low peptide recoveries, analyte losses, and a high time expenditure due to labor-intensive sample pretreatment (review [28]). Therefore, an alternative approach is retaining the analyte on the column by adjusting the chromatographic conditions, as also proposed by Furey et al. (review [28]) and explained in the “Development of the HPLC–MS/MS multimethod” section (LC–MS parameter). As time in the presence of the initial constant eluent composition is increased, an increasing fraction of non-retained matrix components can be eluted before the analyte passes from the column, thus preventing interferences with the ionization process of the analytes themselves. This approach eliminates the need for more sophisticated sample pretreatment or enrichment. Figure 1 illustrates the influence of the focusing time (2, 7, 12, 17, 22 min) on a mixture of CSF and α-factor (1 µM each) in the minimal medium W0 and the mineral medium MV. As a reference, the peptides were dissolved in HPLC-grade water containing 0.125% FA.

After focusing the peptides dissolved in matrix (W0 and MV media) on the column for only 2 min, a loss of signal was observed in both media in comparison to the measurements in HPLC-grade water. The influence on the peak area of α-factor was more pronounced. By extending the focusing time from 2 to 17 min, a steadily increasing signal enhancement was achieved for CSF and α-factor in both culture media (W0 and MV). Peak areas for CSF exceeded the equivalent peak area from the reference in HPLC-grade water. In W0 medium, this point was reached at a focusing time of 12 min (150%), and in MV at 17 min (170%). For α-factor, intensities between 45 and 100% were achieved in W0 media, with 100% being reached after 17 min. In contrast, in MV media, the signals were consistently below the reference, with a maximum of 72% observed after 17 min. The observed signal suppression is likely due to matrix components (composition, see the “Chemicals and materials” section), which could not be sufficiently removed by the focusing approach. This can lead to false-negative results (see review [28]). For instance, inorganic salts are known to cause signal suppression because they are non-volatile as mentioned in the review of Van Eeckhaut et al. (see review [29]). The higher salt content in MV media (due to the presence of MES buffer) could maybe explain the lower peak area compared to that observed for W0 media. In addition, the highly ionizable EDTA (40 mg/L) as a complexing agent can also lead to a reduction in signal intensity as mentioned by Pesek et al. because of the negative charge [30]. Even the vitamins, which are present in higher concentrations in MV media, are eluted very early (almost not retained) and might lead to ion suppression in the region close to the dead volume. Note that the signal enhancement observed (ratio of matrix analyte:reference analyte > 100%) might also be attributable to the presence of matrix components. In addition to the observed signal enhancement with increasing focusing time, an increasing peak width has been observed in both matrix and reference samples. This phenomenon cannot be attributed to media components and is due to diffusion on the column. Also, Furey et al. reviewed the occurrence of broader peptide peaks and reduced sensitivity with increasing analysis time [28].

**Fig. 2:**
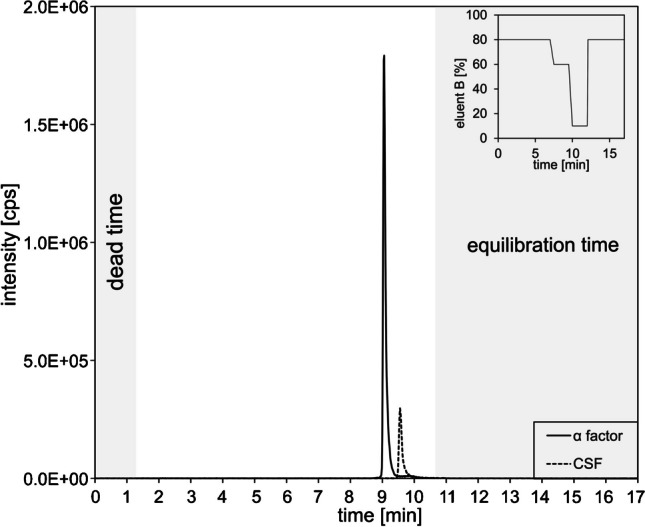
Chromatogram and gradient used for CSF and α-factor using eluent A: 950 mL HPLC-grade water, 5 mL ACN, 1.25 mL FA, 10 mM ammonium formate, and eluent B: 950 mL ACN, 5 mL HPLC-grade water, 1.25 mL FA

Consequently, it is possible to focus the peptides on the column in order to reduce matrix interference. This approach involves the direct use of the sample, without any additional laboratory work or preparation. In consideration of the higher intensities, but longer analysis times and the unfavorable influence on peak widths with long focusing times, a focusing time of 7 min was finally selected. The final gradient is shown in Fig. 2. The peaks reach baseline separation and elute within 10 min. The final 7 min was allotted for the column to reestablish equilibrium for the subsequent run. Nevertheless, an internal standard is used to further correct matrix effects.

#### Investigation of peptide stability in matrices and binding to surfaces

##### Sorption to different vessel surfaces depending on matrix compounds

The stability of peptides can be affected by chemical processes such as oxidation, hydrolysis, arginine conversion, and deamination (see review [31]) and physical effects including surface adsorption, aggregation, and denaturation (see review [32]). All of these processes are dependent, to some extent, on ionic strength [33] and pH (see review [34]). The stability of the signal peptides was investigated in test vessels with different functionalized surfaces (glass, silanized glass, and polypropylene) by adding the synthetic peptide mixture to fresh culture media, HPLC-grade water with 0.125% FA, and HPLC-grade water with 30% ACN and 0.125% FA. Results are shown in Fig. S3 and Fig. S4.

Of the three materials tested, the combination of glass and a 30% proportion of ACN in the sample exhibited the lowest sorption for the tested peptides (including P-factor). The addition of ACN minimizes hydrophobic interactions and a minimum ACN content of 30% was necessary to maintain constant concentrations of all peptides. Other authors [35–37] confirmed the positive influence of higher ACN levels in terms of improved reproducibility and avoidance of adsorption.

Subsequent measurements were carried out using glass vials in conjunction with the addition of ACN. This approach reduces loss due to sorption of the peptides sufficiently and is appropriate for sample volumes of at least 10 µL.

##### Stability in the supernatant of yeast cultivations

**Fig. 3:**
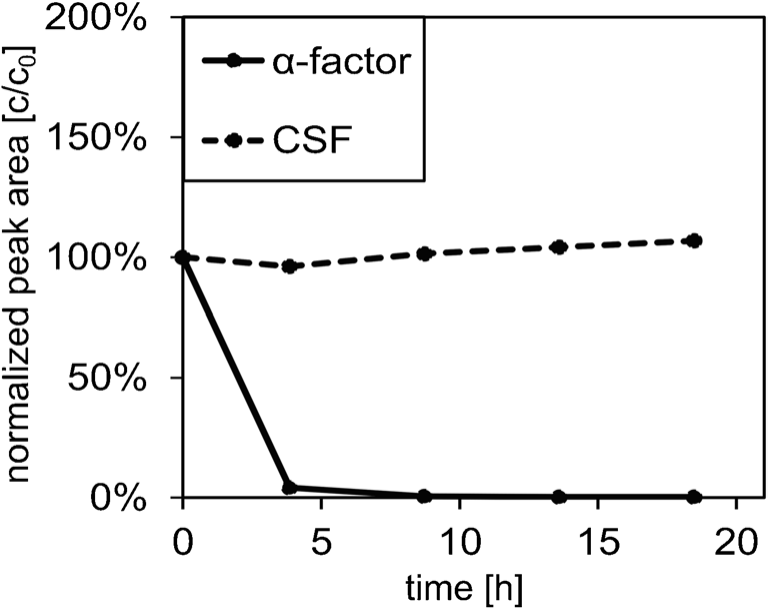
Investigation of peptide stability of the peptide mix (CSF, α-factor) in MV media normalized to initial concentration. The stability was analyzed in the supernatant (after 24 h) of the cultivated yeast control strain. Therefore, the supernatant was spiked with signaling peptide to a final concentration of 1 µM. The remaining peak area was detected over 18 h including several analysis points, *n* = 1

The stability of the peptides also depends on their rate of enzymatic conversation through the action of proteases or peptidases (see review [38]) secreted by cells during the cultivation. Therefore, the concentration of a peptide mixture (containing both α-factor and CSF) was monitored over a period of at least 18 h in the presence of fresh culture media or the supernatant of cultured yeast cells (harvested after 24 h). The results are presented in Fig. 3 (for supernatant from spent MV media), Fig. S5 (in fresh media), and Fig. S6 (for supernatant from spent W0 media).

**Fig. 4:**
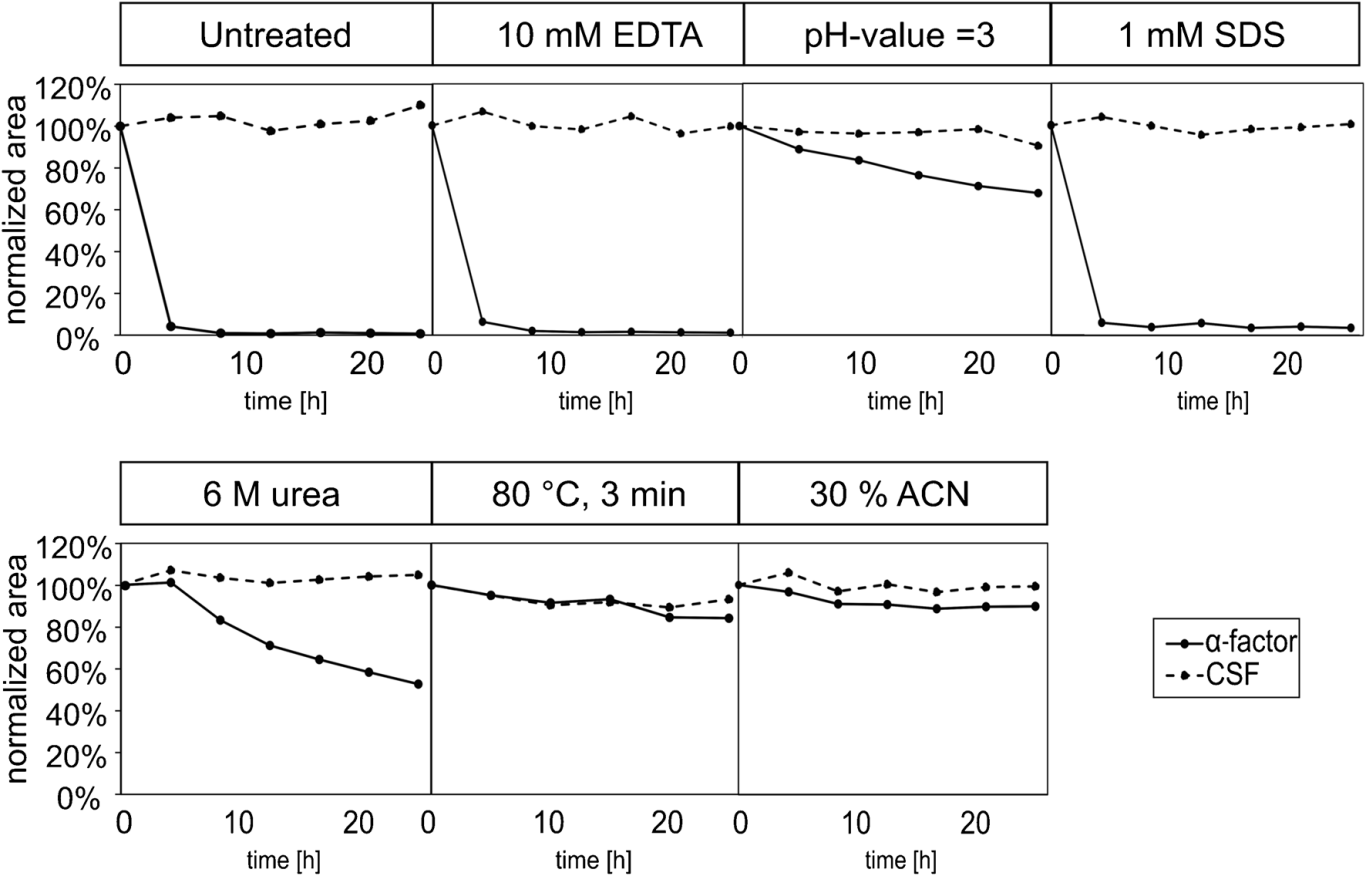
Investigation of the stability of α-factor and CSF, in MV media, in the presence of various factors. Factors tested included the presence of 10 mM EDTA, pH = 3, 1 mM SDS, 6 M urea, 80 °C for 3 min, and 30% ACN. The supernatant of yeast cultivation in MV media was collected after 24 h, spiked with α-factor and CSF, and peptide concentration was monitored for a further 24 h. The peak area was normalized to the initial value. The final peptide concentration was 1 µM for each peptide (*n* = 1)

In fresh culture media (W0 pH = 5.6 or MV pH = 6.5, Fig. S5), the concentration of CSF and α-factor remained nearly constant over 18 h. No evidence of chemical or physical instability of the peptides due to the media components was observed, and the pH value remained constant. Even in the supernatant of the yeast culture in W0 media, CSF remains constant (Fig. S6). In contrast, yeast-specific α-factor was unaffected only in W0 media (Fig. S6), whereas its concentration decreased in MV media, with an almost complete elimination observed within the first 3 h (Fig. 3). This could be attributed to the alterations in pH during the cultivation process. In the unbuffered W0 media, the pH decreases to 2.7 due to decomposition of organic substances—via respiration and the associated formation of carbon dioxide, whereas in the buffered MV media, a final pH of 5.3 was measured. There are a number of potential explanations for the influence of pH on stability of α-factor. On the one hand, the different pH could affect the stability of peptide itself. On the other hand, the activity of the extracellular proteases secreted by *S. cerevisiae* during cultivation (see review [39]) could be altered by pH, as has been observed for other enzymes [40–42], thus influencing the degradation of α-factor. Several approaches have been described in the literature to inactivate degrading enzymes, such as the use of EDTA [43], pH change [44], addition of the denaturant SDS (summarized in [45]), urea [46], organic solvents (see review by [47]), or temperature deactivation [48], all of which have been applied in this study to investigate their impact on the stability of α-factor in the supernatants of spent yeast cultures (Fig. 4).

In the untreated sample, the concentration of the bacterial peptide CSF remains stable over 24 h, while the α-factor concentration declines to nearly zero within the first 4-h interval. The different treatment methods have no negative effect on CSF levels for which consistently stable concentrations (above 90% remaining) were observed. However, for α-factor, EDTA and SDS have no effect on stability. Changing the pH to 3 leads to an improved stability with a loss of only 30% and the addition of urea leads to a loss of 50% over time. Thermal treatment (heating to 80 °C for 3 min) or the addition of 30% ACN to the sample resulted in near stable α-factor concentrations. The results indicate that adjusting the sample to a 30% ACN content would be beneficial in terms of stability and sorption (as shown in Fig. S4). In subsequent studies (see the “Transferability of the quantification method to P-factor of *S. pombe* in EMM media” section), the method was extended for P-factor, which required a concentration of 50% ACN to reduce adsorption to the glass. This level of ACN was chosen for further analyses.

The final LC and MS parameters for peptide measurements are presented in Table 4.

**Table 4:** Optimized LC and MS conditions for the validation and peptide analysis including all previously obtained results

Column	HILIC column TSK Gel Amide 80	Gradient		
Eluent A	950 mL HPLC-grade water 50 mL ACN 1.25 mL FA 10 mM NA	Time (min)	Vol.% Eluent A	Vol.% Eluent B
		0	20	80
		7	20	80
		7.5	40	60
		9.5	40	60
		10	90	10
		12	90	10
		12.1	20	80
		17	20	80
Eluent B	50 mL HPLC-grade water 950 mL ACN 1.25 mL FA			
Flow rate	0.4 mL/min			
Sample vessel	Glass vial			
Injection volume	10 µL			
T (ion source)	550 °C	Sample preparation • Mixing 50% supernatant with 50% ACN until a final volume of 150 µL • Adding 10 µL ISTD • Analysis using LC–MS/MS 6500 +		
Gas 1, 2	50 psi			
CAD gas	Medium			
Curtain gas	40 psi			
Voltage	+ 5500 V			

#### Validation

The LC–MS/MS method was validated using the optimized conditions (see Table 4). Thus, the yeast supernatant was mixed in a 1:1 ratio with ACN and analyzed. The values presented in Table 5 were determined using the procedures described in the methods section. Raw data and further calculated data are in the SI (Tables S2–S7).

**Table 5:** Validation results of the peptide quantification method for α-factor and CSF. Calculation of the linearity, precision, robustness, LOD and LOQ (over signal to noise ratio)

Parameter	Peptide	Value				
Calibration line (0.01–1 µM)	α-Factor	*y* = 2.5860*x* − 0.0088; *R* = 0.9994				
	CSF	*y* = 1.1703*x* − 0.0088; *R* = 0.9993				
Precision (RSD)	Concentration	0.05 µM		0.2 µM		0.7 µM
	α-Factor (%)	5.1		4.1		1.2
	CSF (%)	4.6		4.8		1.2
Robustness (0.2 µM, RSD)	Matrix	H_2_O	MV	Yeast MV	W0	Yeast W0
	α-Factor (%)	1.6	1.1	0.3	1.4	0
	CSF (%)	3.9	1.5	0.9	0.6	0.3
LOD	α-Factor	LOD = 0.01 µM				
	CSF	LOD = 0.02 µM				
LOQ	α-Factor	LOQ = 0.03 µM				
	CSF	LOQ = 0.05 µM				

Finally, an LOD of 0.01 µM for α-factor and 0.02 µM for CSF was achieved. In comparison to the findings of Hennig et al., a 100-fold lower LOD for α-factor (LOD = 0.1 nM) was obtained using a modified ELISA test [22]. ELISA has a higher sensitivity than the LC–MS/MS method presented here, but has lower sample throughput and longer analysis times and cannot specify between different peptides. Furthermore, the immunological system utilized is susceptible to alterations in pH and media composition, as previously highlighted by Hennig et al. [22]. Additionally, antibodies are not available for all substances and the antibody must be immobilized on a surface, with the orientation of the antibody influencing the sensitivity of the assay. The β-galactosidase assay has been demonstrated to detect CSF concentrations as low as 0.05 nM [19]. This is considerably lower than the method presented here, but the measurement of β-galactosidase activity is time-consuming and the establishment of reporter cells for the uptake of the signal peptide is also needed. Moreover, modifying the matrix composition or introducing additional substances is not straightforward. Furthermore, both methods are unable to quantify multiple peptides simultaneously, which is a critical limitation, particularly in the context of cross-kingdom communication. Whereas in the present study, the analysis of a peptide mix in matrix requires only a 10 µL volume, with results available within 17 min. Furthermore, the LC–MS/MS method employed here allows for the simultaneous analysis of multiple peptides without the necessity for reporter cells or antibodies.

### Case study: measurement of real samples

Here, the methodology for quantifying the signaling peptides developed in this study is tested under co-cultivation conditions as a prerequisite for cross-kingdom communication. To this end, the yeast *S. cerevisiae* was genetically modified to secrete both its own α-factor and the bacteria-specific CSF into the surrounding culture media during cultivation. The secreted CSF peptide should be recognized by the bacterium *B. subtilis*. In earlier investigations [17], α-factor secretion was only identified qualitatively by the “Shmoo effect” and CSF by luminescence using the luciferase assay or fluorescence. The newly developed LC–MS/MS method allows for direct and simultaneous quantification of both peptides in a matrix. Since the amount of peptide produced depends on the cultivation time and the culture media [49], peptide quantification was performed for minimal W0 medium and mineral MV medium (latter used as co-cultivation media as that is suitable for both *S. cerevisiae* and *B. subtilis*) at different time points.

**Fig. 5:**
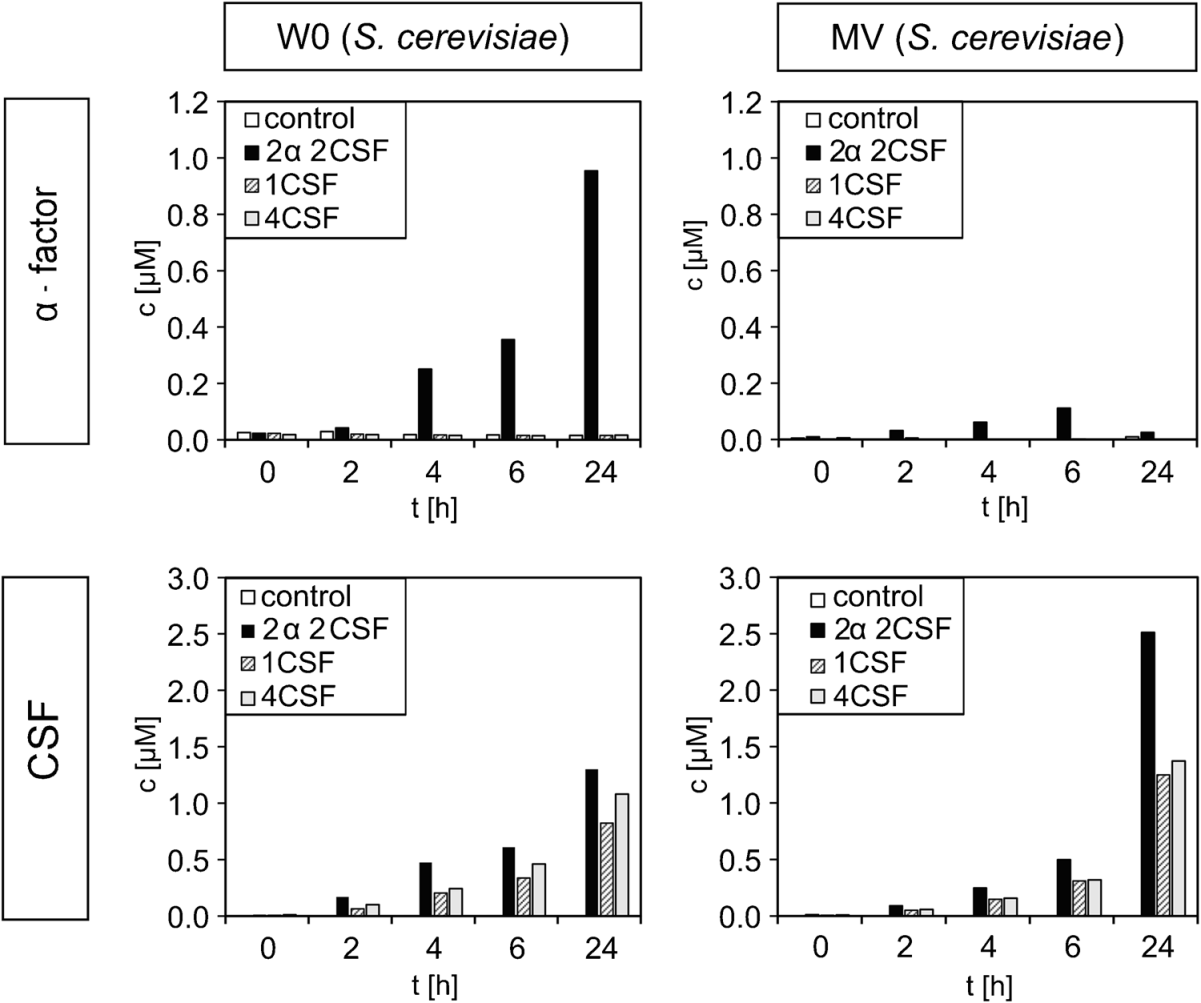
Measurements of the secreted α-factor and CSF peptides from *S. cerevisiae* BY4717Δ*bar1* in W0 and MV media. Yeast strain BY4717Δ*bar1* was cultivated harboring two copies of each of the genes encoding α-factor and CSF (2α2CSF); one single gene encoding CSF (1CSF); and four copies of the genes encoding CSF (4CSF), respectively. Samples were taken after 0, 2, 4, 6, and 24 h and directly mixed 1:1 with ACN. Afterwards, an ISTD was added prior to analysis

The secreted peptides in the supernatant of the cultured yeasts in W0 and MV media were analyzed and the results are shown in Fig. 5.

Both α-factor and CSF were successfully detected in both culture media (see Fig. 5), although at different concentrations. Concurrently, the peptide concentration increased with increasing cultivation time and cell growth. This resulted in CSF concentrations of 1.2 µM (24 nmol) in W0 and 2.5 µM (50 nmol) in MV media after 24 h. Surprisingly, the number of copies of the respective genes carried on the plasmid had no effect on expression levels (see Table 2), as supernatants of strain 2α2CSF contained higher levels of CSF than 4CSF in both W0 and MV media. This was also confirmed by Vološen et al. [17]. The higher CSF concentration in MV medium may be attributed to the greater number of cells present. Furthermore, the secretion of CSF was found to be greater than the 100 nM reported in the studies of Lazazzera et al. and Solomon et al.—using a wild-type *B. subtilis* and a β-galactosidase assay to measure CSF concentration [16, 19]. With regard to α-factor, a concentration of 0.95 µM (19 nmol) was observed in the W0 medium after 24 h, while a concentration of only 0.1 µM (2 nmol) α-factor was found in the MV medium after 6 h and 0.025 µM (0.5 nmol) after 24 h. Under natural, non-overexpressing conditions, *S. cerevisiae* has been observed to secrete α-factor in the range of 30–50 nM within 10 h [22] or up to 20 nM [49], whereas the secreted α-factor concentration increases with higher population density [49]. The concentration of α-factor can be in the range of 1–2 µM within 10 h if using a genetically modified GPD promoter in combination with the α-factor gene in yeast cells—as reported by Hennig et al. [22]. The amount of α-factor present in the W0 medium is comparable to the value determined in the literature [22] using the *FIG1* promoter. The low concentration of α-factor in MV medium is potentially caused by the lower enzymatic stability of α-factor in this medium, as was previously observed in the stability measurements (Fig. 3).

### Transferability of the quantification method to P-factor of *S. pombe* in EMM media

**Fig. 6:**
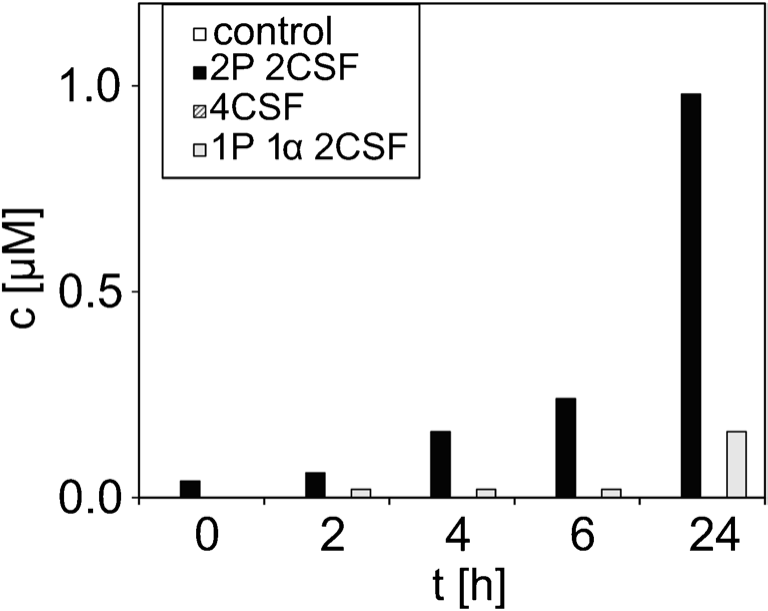
Quantification of secreted P-factor in the supernatant of *S. pombe* in EMM media during a 24-h cultivation period (*n* = 1). *S. pombe* HE620 was cultivated carrying a construct of two copies of each gene encoding for P-factor and CSF (2P2CSF), four copies of CSF (4CSF) and one copy of the genes encoding for P- and α-factor, and two copies encoding for CSF (1P1α2CSF). Samples were taken after 0, 2, 4, 6, and 24 h and directly mixed 1:1 with ACN. An ISTD was added prior to analysis

Next, the quantification method was applied to another important pheromone: the P-factor of *S. pombe*. This pheromone is similar in terms of functionality to the α-factor of *S. cerevisiae*, but has a larger and more complex structure (see Table 1). The method developed and validated in this course of this study was extended to this pheromone, with a LOD and LOQ of 0.01 µM and 0.02 µM. Also, the peptide stability in the typical EMM media used for *S. pombe* was demonstrated (see Fig. S7). Further validation results can be found in Table S8. The results are presented in Fig. 6.

With increasing cultivation time, the concentration of P-factor in the supernatant increased. After 24 h, a concentration of 1 µM (20 nmol) was achieved in *S. pombe* HE620 2P2CSF and 0.16 µM (3.2 nmol) from the plasmid with only one gene that encodes for P-factor. No P-factor was detected in the control sample and from the sample carrying the 4CSF construct. In previous work, Hennig et al. only demonstrated the presence of P-factor qualitatively through morphological changes, fluorescence measurements, and SDS-PAGE analysis [50]. According to Hennig et al., more P-factor than the required minimal 10 nM P-factor for cell response is produced through genetic modification of the yeast *S. pombe* [50].

Finally, the method developed and validated here can be transferred to other yeast strains and pheromones as well as other cultivation media.

## Conclusion

The methods commonly used for the determination of pheromones are time-consuming, imprecise, or limited to single peptides or require the addition of certain reagents. The analytical method presented here can be used for the simultaneous measurement of yeast (α-factor, P-factor)- and bacteria (CSF)-specific peptides in different matrices (W0, EMM, and MV media) without pre-concentration and enabling high-throughput analyses. The three selected peptides, differing in polarity and size according to their amino acid sequences, were analyzed using a TSKgel HILIC separation column. Using 0.125% FA, in both eluents, and 10 mM ammonium formate in the aqueous eluent a simultaneous measurement of the synthetic peptides was possible. The optimized conditions resulted in a signal peptide detection method with an LOD of 10 nM for α- and P-factor and 20 nM for CSF. In contrast, a lower LOD was achieved by Hennig et al. for α-factor using an ELISA test [22], and by Lazazzera et al. for CSF by measuring β-galactosidase activity [19]. However, both methods are time-consuming and limited to the measurement of one peptide, rendering them unsuitable for this assay. If required, the LOD and LOQ of the LC–MS/MS can be reduced maybe by increasing the injection volume, lowering the flow rate, changing the dimension of the column, or using SPE to pre-concentrate the peptide, for example, by a factor of 160 [51] or 100 [52]. The maximum concentrations of secreted peptides that were detected in the matrix were 1 µM for α- and P-factor and 2.5 µM for CSF under simultaneous measurement conditions. Consequently, the genetic modification and the amplification of the amount of secreted peptides were successful.

Finally, the results show that the developed method can be used to simultaneously detect and quantify various secreted peptides. The possibility for direct measurements, with the necessity of only small volumes and without the need for sample pretreatment, is a great advantage in signal peptide quantification. Furthermore, this validated method is easily transferable to other laboratories while the more sensitive ELISA is not commercially available. As the measurements are relatively short (compared to ELISA), it is possible to track signaling peptides in near-real time, which will advance research in cross-kingdom communication.

## Supplementary Information

Below is the link to the electronic supplementary material.

Supplementary file1 (DOCX 2966 KB)

## Data Availability

The data is available in the main manuscript and in the supplementary file if mentioned.
