# Supplementary material for: LC–MS/MS quantification of bacterial and fungal signal peptides via direct injection: a case study of cross-kingdom communication
Source: Anal Bioanal Chem. 2025 Feb 4;417(8):1677–89. doi: 10.1007/s00216-025-05767-6 (PMC11876276; doi:10.1007/s00216-025-05767-6)
Supplement: Supplementary file 1 — Supplementary file1 (DOCX 2966 KB) [file 216_2025_5767_MOESM1_ESM.docx]

Supplementary Information

LC-MS/MS Quantification of bacterial and fungal signal peptides via direct injection: A case study of cross-kingdom communication

Carolin Pohl^1^[^0009-0009-1150-3133^], Linda Schuster^1^, Cindy Rau^1,2^, Uta Gutbier^3,4^, Stephan Beil^1^, Hilmar Börnick^1^, Kai Ostermann^4^ and Stefan Stolte^1^[0000-0001-5186-3955]

^1^ Faculty of environmental science, Institute of Water Chemistry, TUD Dresden University of Technology, 01062 Dresden, Germany

^2^ Faculty of Civil Engineering, Division of Water Sciences, HTW University of Applied Sciences, Friedrich-List-Platz 1, 01069 Dresden, Germany

^3^ Else Kröner Fresenius Center for Digital Health, Faculty of Medicine Carl Gustav Carus, TUD Dresden University of Technology, Dresden, Germany

^4^ Faculty of Biology, Research Group Biological Sensor-Actuator-Systems, TUD Dresden University of Technology, 01062 Dresden, Germany
[stefan.stolte@tu-dresden.de](mailto:stefan.stolte@tu-dresden.de)

### For compound tuning, 1 µM synthetic α- or P-factor or 2 µM CSF were dissolved in 70 % ACN and 30 % LC-MS water + 0.125 % FA and injected directly into the MS instrument. With the integrated automated tuning set up, the precursor ion was determined through the declustering potential (DP) and entrance potential (EP). For the product ion the Collision Energy (CE) and Cell Exit Potential (CXP) were optimized for the six highest intensity transitions. The two best transitions (Quantifier and Qualifer) were included in the final MRM-method.

**Table S1.** MS Transitions for the tandem mass spectrometer QTRAP^®^6500^+^ of Sciex, EP 10 V, positive ionisation method and the corresponding retention time

| peptide | Q1 (m/z) | Q3 (m/z) | Retention time | DP (V) | CE (V) | CXP (V) |
| --- | --- | --- | --- | --- | --- | --- |
| **α factor** | 842.6 | **324.0** | **9.06** | **145** | **47** | **36** |
|  | 842.6 | **510.1** | **9.06** | **145** | **49** | **22** |
| **CSF** | 593.1 | **492.2** | **9.58** | **36** | **43** | **24** |
|  | 593.1 | **267.0** | **9.58** | **36** | **49** | **30** |
| **P-factor** | 930.5 | **825.4** | **8.89** | **91** | **45** | **22** |
|  | 930.5 | **711.4** | **8.89** | **91** | **49** | **14** |
| **α-factor ISTD** | 849.1 | **324.1** | **9.06** | **145** | **49** | **36** |
|  | 849.1 | **510.1** | **9.06** | **145** | **49** | **22** |
| **CSF ISTD** | 603.1 | **502.1** | **9.58** | **111** | **45** | **22** |
|  | 603.1 | **317.0** | **9.58** | **111** | **49** | **14** |
| **P-factor ISTD** | 936.0 | **825.5** | **8.89** | **106** | **43** | **36** |
|  | 936.0 | **711.3** | **8.89** | **106** | **45** | **32** |


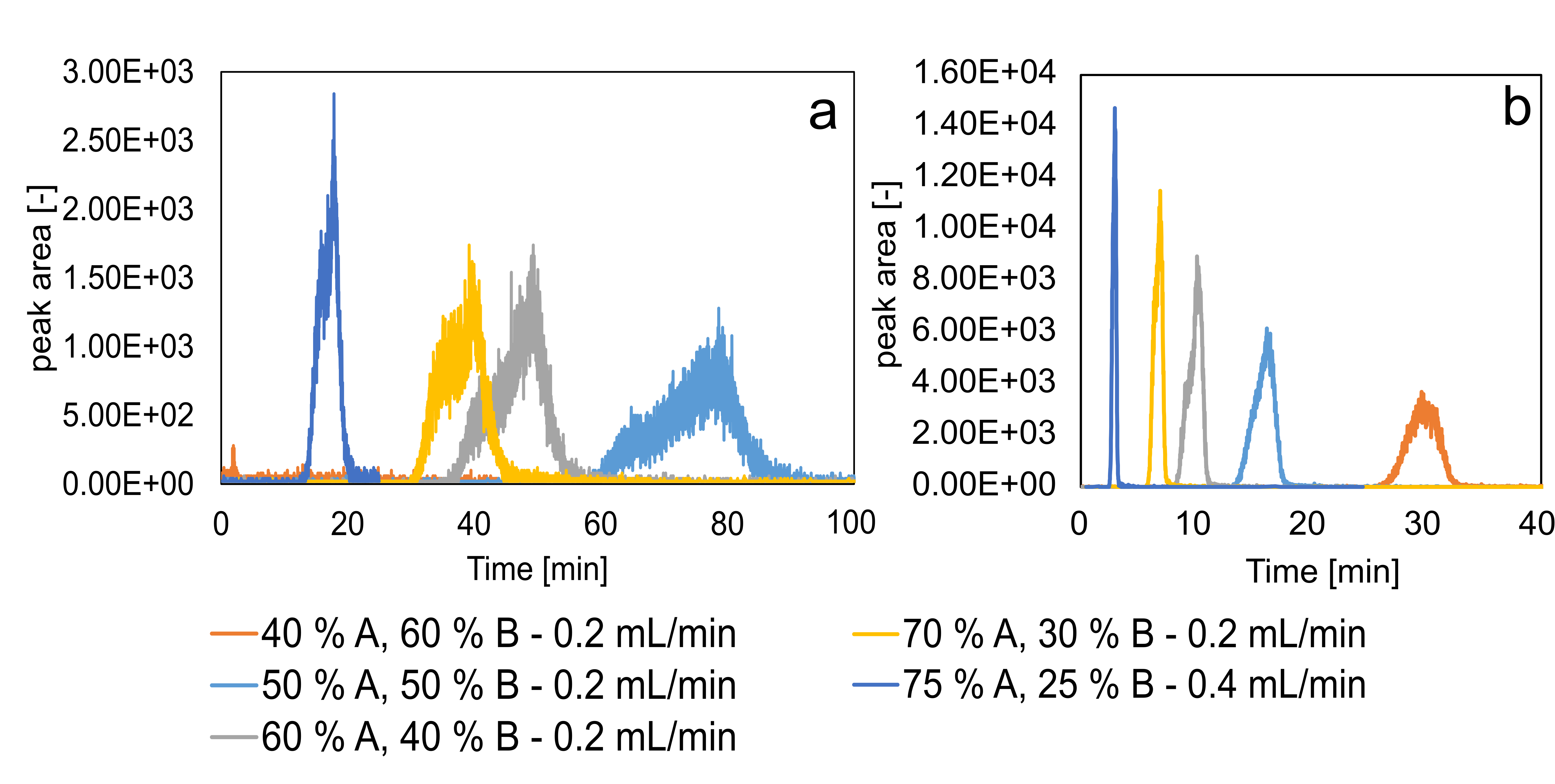


**Fig. S1.** Chromatogram of α-factor (a) and CSF (b) elution using isocratic eluent composition on the LC-MS/MS 3200 QTRAP. Eluent A consists of HPLC grade water with 0.1 % CH_3_COOH and eluent B consists of ACN + 0.1 % CH_3_COOH. The flow rate was 0.2 -0.4 mL/min. The proportion of eluent A was varyied between 40 – 75 %.


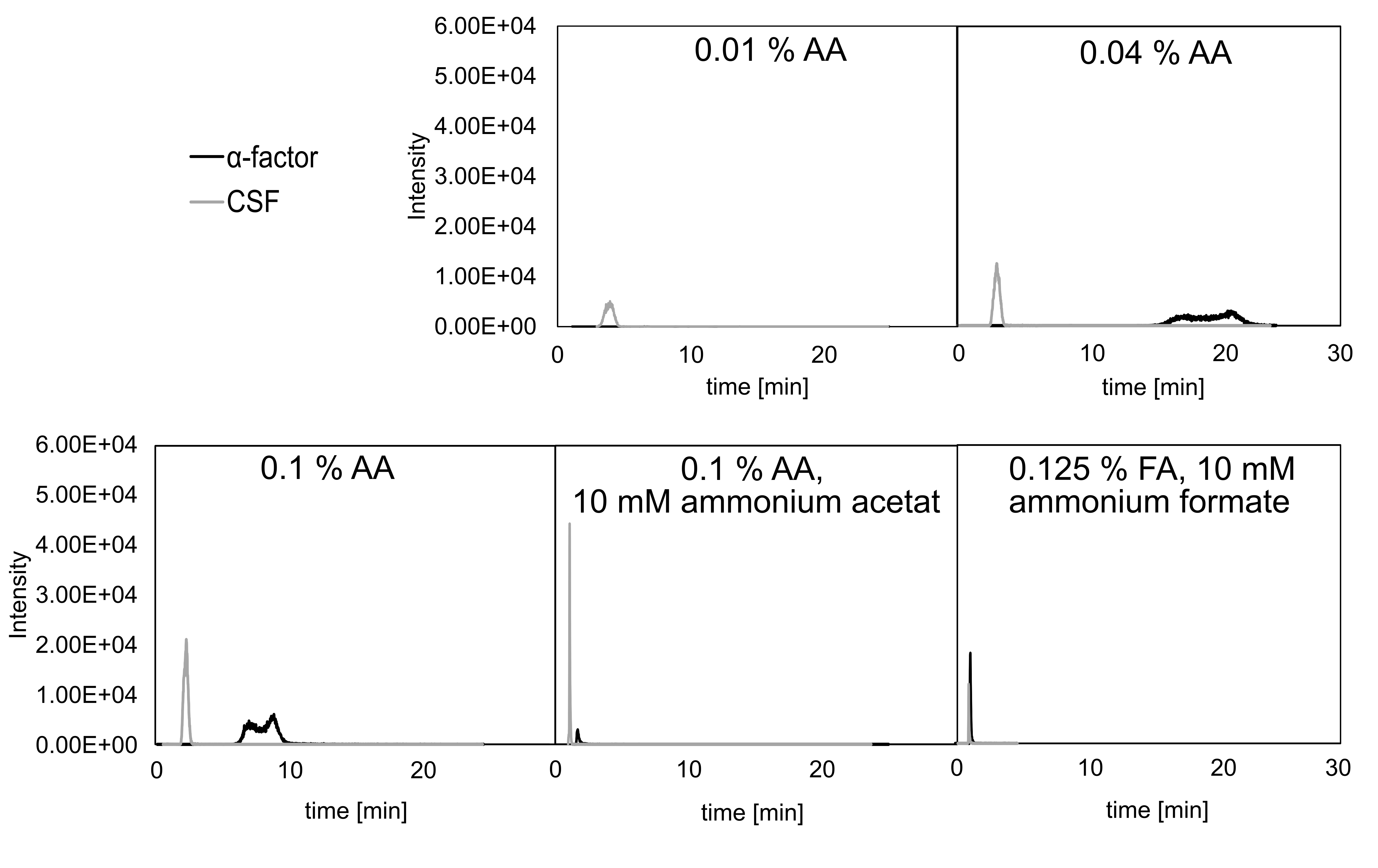


**Fig. S2.** Peptide peaks for α-factor and CSF as a function of acid (acetic acid - AA and formic acid - FA), acid concentration (0.01, 0.04, 0.1 and 0.125 %) and buffer (10 mM ammonium acetate or 10 mM ammonium formate). Measurements were performed on a LC-MS/MS 3200 QTRAP with isocratic conditions of 75 % HPLC grade water and 25 % ACN. The overall duration was 30 min. A peptide concentration of 1 µM for each peptide was used.


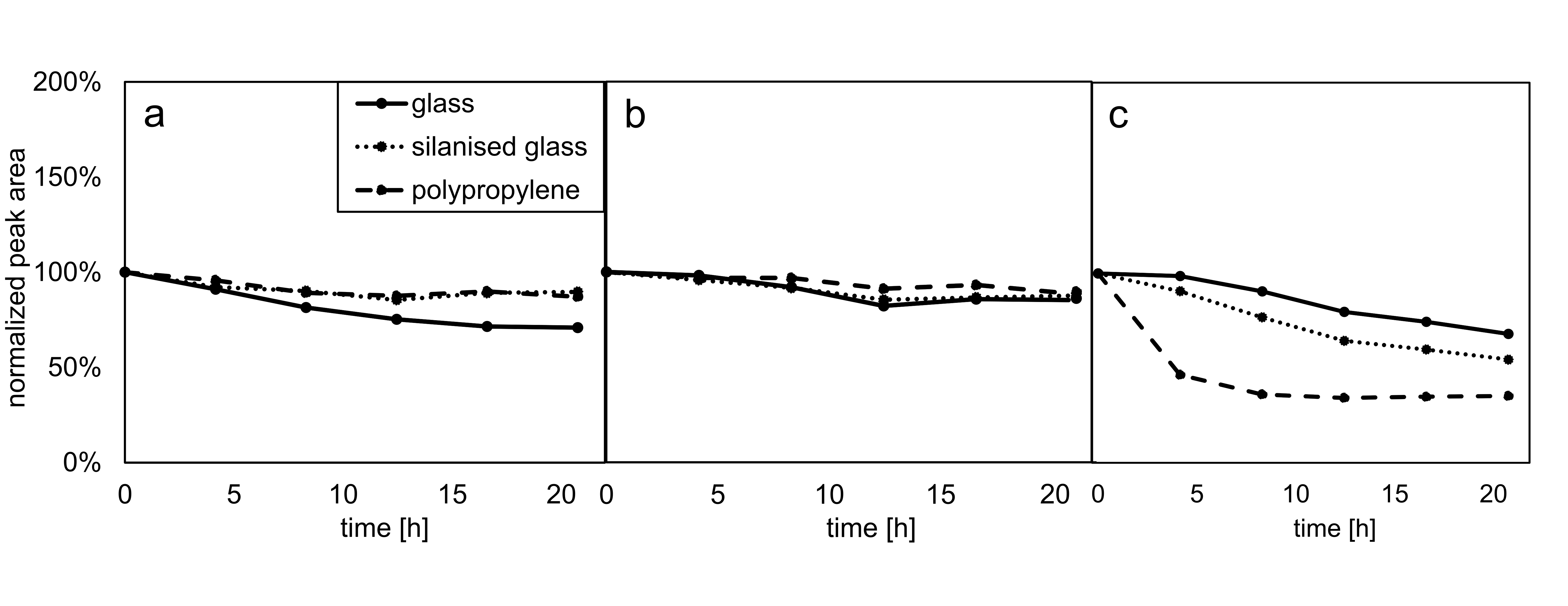


**Fig. S3.** Comparison of the normalized peak area of α-factor (a), CSF (b) and P-factor (c) as a consequence of adsorption to different material surfaces: glass, silanised glass and polypropylene. Peptides were dissolved in LC-MS grade water with 0.125 % FA, pH 2.5. The analysis was conducted over a period of 22 h. A peptide concentration of 1 µM for each peptide was used.


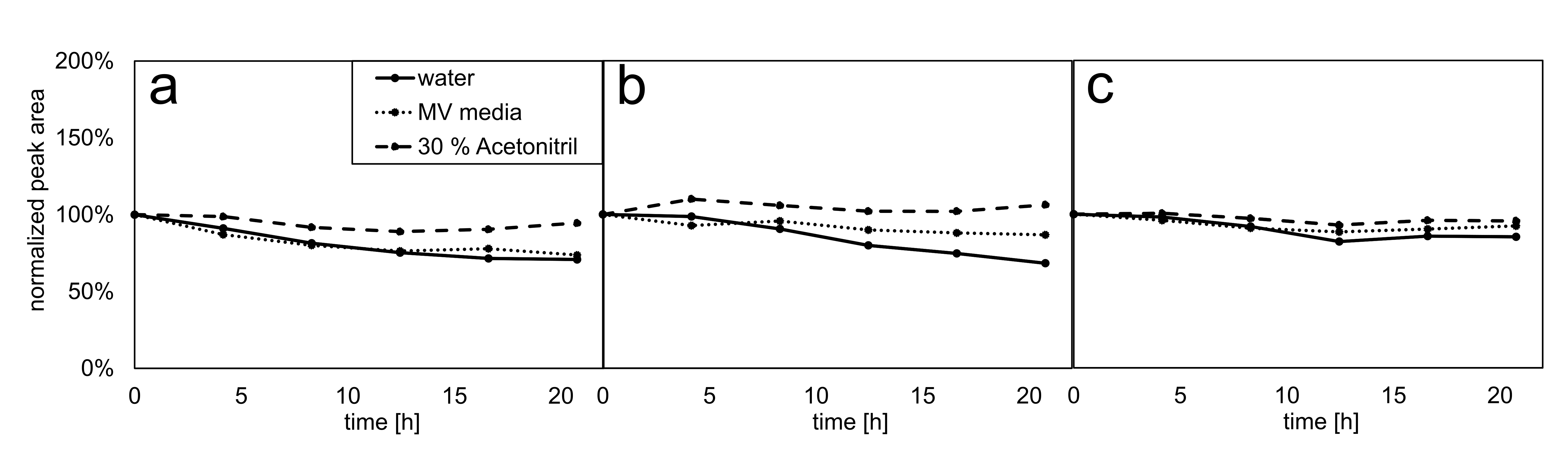


**Fig. S4.** Comparison of the normalized peak area of α- factor (a), CSF (b) and P-factor (c) in glass vessels according to the respective matrix: LC-MS grade water + 0.125 % FA, MV media, LC-MS grade water with 0.125 % FA + 30 % ACN. The analysis was conducted over a period of 22 h. A peptide concentration of 1 µM for each peptide was used.


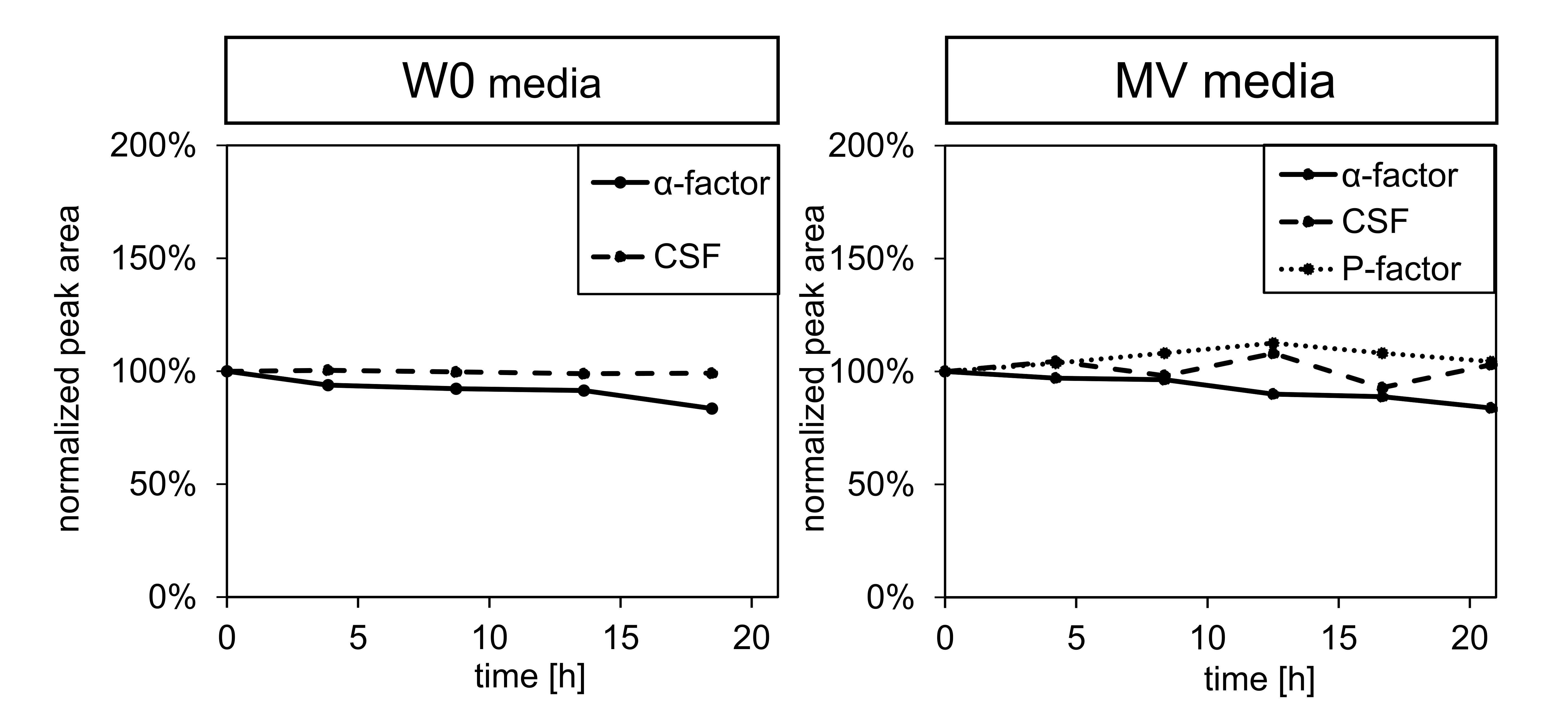


**Fig. S5.** Normalized peak area of CSF and α- factor in glass vessels in fresh W0 and MV-medium (with additional P-factor) conduced over 18 h. A peptide concentration of 1 µM for each peptide was used.


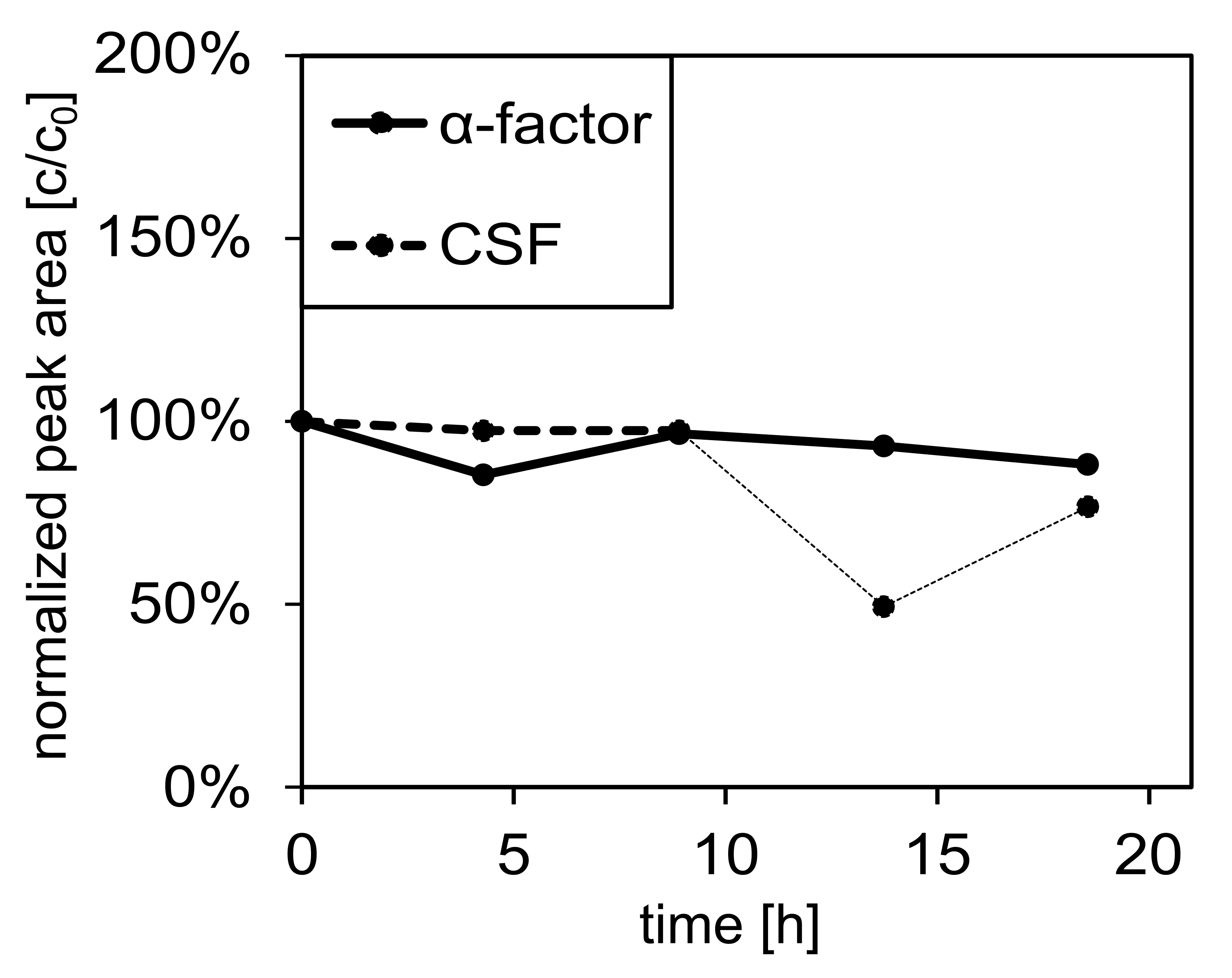


**Fig. S6.** Investigation of the peptide stability of the peptide mix (CSF, α-factor) in W0 media. The stability was analyzed in the supernatant of the cultivated yeast control strain after 24 h, n=1. A peptide concentration of 1 µM for each peptide was used. In the supernatants of the yeast cultures the concentration of CSF appears to decrease in the third time point and to increase until the end. However, this could not be confirmed in a second measurement using another yeast strain, so that it is declared as an outlier

**Table S2.** Values for the calculation of the LOD, LOQ and calibration line (**tandem mass spectrometer QTRAP^®^6500^+^ of Sciex)**

| **Area ratio/**  **peptide** | **Conc. [µM]** | **1** | **2 (repeat of 1)** | **3** | **4 (repeat of 3)** | **5** | **6 (repeat of 5)** | **average** |
| --- | --- | --- | --- | --- | --- | --- | --- | --- |
| **CSF**  **(CSF1_1)** | 0.01 | 7.43E-03 | 8.95E-03 | 8.94E-03 | 8.68E-03 | 1.01E-02 | 1.10E-02 | 9.18E-03 |
|  | 0.02 | 1.96E-02 | 1.63E-02 | 2.16E-02 | 2.00E-02 | 2.40E-02 | 2.22E-02 | 2.06E-02 |
|  | 0.03 | 2.78E-02 | 3.23E-02 | 3.15E-02 | 3.04E-02 | 3.16E-02 | 2.77E-02 | 3.02E-02 |
|  | 0.04 | 4.13E-02 | 3.92E-02 | 4.41E-02 | 4.06E-02 | 4.50E-02 | 4.05E-02 | 4.18E-02 |
|  | 0.05 | 5.14E-02 | 5.07E-02 | 5.34E-02 | 5.76E-02 | 5.53E-02 | 5.53E-02 | 5.40E-02 |
|  | 0.06 | 6.17E-02 | 6.39E-02 | 6.17E-02 | 6.41E-02 | 7.18E-02 | 6.63E-02 | 6.49E-02 |
|  | 0.07 | 6.98E-02 | 7.42E-02 | 6.99E-02 | 7.07E-02 | 8.00E-02 | 7.58E-02 | 7.34E-02 |
|  | 0.08 | 8.52E-02 | 8.31E-02 | 7.46E-02 | 8.32E-02 | 9.16E-02 | 8.58E-02 | 8.39E-02 |
|  | 0.09 | 9.84E-02 | 9.73E-02 | 1.10E-01 | 8.84E-02 | 1.06E-01 | 1.01E-01 | 1.00E-01 |
|  | 0.1 | 1.11E-01 | 9.84E-02 | 1.07E-01 | 1.08E-01 | 1.17E-01 | 1.09E-01 | 1.08E-01 |
|  | 0.2 | 1.13E-01 | 2.05E-01 | 2.24E-01 | 2.13E-01 | 2.41E-01 | 2.15E-01 | 2.02E-01 |
|  | 0.3 | 3.72E-01 | 3.24E-01 | 3.33E-01 | 3.17E-01 | 3.77E-01 | 3.59E-01 | 3.47E-01 |
|  | 0.4 | 4.92E-01 | 4.36E-01 | 4.69E-01 | 4.20E-01 | 4.85E-01 | 4.77E-01 | 4.63E-01 |
|  | 0.5 | 5.34E-01 | 5.34E-01 | 5.56E-01 | 5.20E-01 | 6.09E-01 | 5.94E-01 | 5.58E-01 |
|  | 0.6 | 6.66E-01 | 6.57E-01 | 6.89E-01 | 6.48E-01 | 7.25E-01 | 6.98E-01 | 6.80E-01 |
|  | 0.7 | 7.77E-01 | 7.57E-01 | 7.99E-01 | 7.56E-01 | 8.65E-01 | 8.91E-01 | 8.07E-01 |
|  | 0.8 | 9.21E-01 | 8.61E-01 | 9.34E-01 | 8.89E-01 | 9.93E-01 | 9.39E-01 | 9.23E-01 |
|  | 0.9 | 1.12E+00 | 9.77E-01 | 9.82E-01 | 9.70E-01 | 1.14E+00 | 1.07E+00 | 1.04E+00 |
|  | 1 | 1.14E+00 | 1.20E+00 | 1.12E+00 | 1.14E+00 | 1.36E+00 | 1.16E+00 | 1.19E+00 |

**Table S3.** Values for the calculation of the LOD, LOQ and calibration line (**tandem mass spectrometer QTRAP^®^6500^+^ of Sciex)**

| **Area ratio/**  **peptide** | **Conc. [µM]** | **1** | **2 (repeat of 1)** | **3** | **4 (repeat of 3)** | **5** | **6 (repeat of 5)** | **average** |
| --- | --- | --- | --- | --- | --- | --- | --- | --- |
| **α-factor (MF2_1)** | 0.01 | 2.62E-02 | 2.94E-02 | 3.11E-02 | 3.38E-02 | 2.82E-02 | 2.88E-02 | 2.96E-02 |
|  | 0.02 | 5.18E-02 | 5.49E-02 | 5.68E-02 | 5.52E-02 | 5.05E-02 | 4.80E-02 | 5.29E-02 |
|  | 0.03 | 6.96E-02 | 7.41E-02 | 7.51E-02 | 7.84E-02 | 7.32E-02 | 7.40E-02 | 7.41E-02 |
|  | 0.04 | 1.05E-01 | 1.06E-01 | 1.01E-01 | 1.07E-01 | 9.77E-02 | 1.08E-01 | 1.04E-01 |
|  | 0.05 | 1.19E-01 | 1.29E-01 | 1.26E-01 | 1.44E-01 | 1.23E-01 | 1.26E-01 | 1.28E-01 |
|  | 0.06 | 1.48E-01 | 1.55E-01 | 1.51E-01 | 1.58E-01 | 1.49E-01 | 1.55E-01 | 1.52E-01 |
|  | 0.07 | 1.61E-01 | 1.79E-01 | 1.77E-01 | 1.87E-01 | 1.71E-01 | 1.76E-01 | 1.75E-01 |
|  | 0.08 | 2.07E-01 | 2.09E-01 | 1.86E-01 | 2.12E-01 | 1.97E-01 | 1.98E-01 | 2.01E-01 |
|  | 0.09 | 2.22E-01 | 2.26E-01 | 2.51E-01 | 2.30E-01 | 2.15E-01 | 2.39E-01 | 2.30E-01 |
|  | 0.1 | 2.49E-01 | 2.55E-01 | 2.48E-01 | 2.58E-01 | 2.41E-01 | 2.57E-01 | 2.51E-01 |
|  | 0.2 | 2.48E-01 | 4.85E-01 | 5.18E-01 | 5.24E-01 | 4.91E-01 | 4.98E-01 | 4.61E-01 |
|  | 0.3 | 8.26E-01 | 7.97E-01 | 7.42E-01 | 7.65E-01 | 7.32E-01 | 7.85E-01 | 7.75E-01 |
|  | 0.4 | 1.06E+00 | 1.02E+00 | 1.03E+00 | 1.05E+00 | 1.01E+00 | 1.03E+00 | 1.03E+00 |
|  | 0.5 | 1.19E+00 | 1.28E+00 | 1.29E+00 | 1.29E+00 | 1.20E+00 | 1.28E+00 | 1.25E+00 |
|  | 0.6 | 1.43E+00 | 1.56E+00 | 1.52E+00 | 1.56E+00 | 1.46E+00 | 1.53E+00 | 1.51E+00 |
|  | 0.7 | 1.70E+00 | 1.70E+00 | 1.78E+00 | 1.83E+00 | 1.75E+00 | 1.99E+00 | 1.79E+00 |
|  | 0.8 | 2.05E+00 | 2.03E+00 | 2.03E+00 | 2.15E+00 | 2.00E+00 | 2.06E+00 | 2.05E+00 |
|  | 0.9 | 2.49E+00 | 2.35E+00 | 2.23E+00 | 2.32E+00 | 2.27E+00 | 2.25E+00 | 2.32E+00 |
|  | 1 | 2.46E+00 | 2.77E+00 | 2.41E+00 | 2.81E+00 | 2.77E+00 | 2.57E+00 | 2.63E+00 |

**Table S4.** Values for the calculation of the LOD, LOQ and calibration line (**tandem mass spectrometer QTRAP^®^6500^+^ of Sciex)**

| **Area ratio/**  **peptide** | **Conc. [µM]** | **1** | **2 (repeat of 1)** | **3** | **4 (repeat of 3)** | **5** | **6 (repeat of 5)** | **average** |
| --- | --- | --- | --- | --- | --- | --- | --- | --- |
| **P-factor (P3_1)** | 0.01 | 3.25E-02 | 3.27E-02 | 3.39E-02 | 3.88E-02 | 2.86E-02 | 2.69E-02 | 3.23E-02 |
|  | 0.02 | 6.59E-02 | 6.24E-02 | 6.36E-02 | 5.71E-02 | 4.90E-02 | 3.92E-02 | 5.62E-02 |
|  | 0.03 | 8.32E-02 | 9.07E-02 | 8.57E-02 | 8.14E-02 | 6.62E-02 | 6.37E-02 | 7.85E-02 |
|  | 0.04 | 1.19E-01 | 1.24E-01 | 1.13E-01 | 1.12E-01 | 9.00E-02 | 8.23E-02 | 1.07E-01 |
|  | 0.05 | 1.43E-01 | 1.57E-01 | 1.45E-01 | 1.63E-01 | 1.12E-01 | 1.03E-01 | 1.37E-01 |
|  | 0.06 | 1.83E-01 | 1.81E-01 | 1.73E-01 | 1.70E-01 | 1.34E-01 | 1.36E-01 | 1.63E-01 |
|  | 0.07 | 2.05E-01 | 2.11E-01 | 1.96E-01 | 2.01E-01 | 1.51E-01 | 1.52E-01 | 1.86E-01 |
|  | 0.08 | 2.38E-01 | 2.37E-01 | 1.97E-01 | 2.30E-01 | 1.71E-01 | 1.71E-01 | 2.07E-01 |
|  | 0.09 | 2.78E-01 | 2.71E-01 | 2.93E-01 | 2.59E-01 | 1.87E-01 | 1.99E-01 | 2.48E-01 |
|  | 0.1 | 2.95E-01 | 2.96E-01 | 2.84E-01 | 2.94E-01 | 2.23E-01 | 2.05E-01 | 2.66E-01 |
|  | 0.2 | 3.02E-01 | 6.09E-01 | 5.88E-01 | 5.71E-01 | 4.18E-01 | 4.30E-01 | 4.86E-01 |
|  | 0.3 | 9.94E-01 | 9.54E-01 | 8.31E-01 | 8.26E-01 | 6.29E-01 | 6.43E-01 | 8.13E-01 |
|  | 0.4 | 1.34E+00 | 1.23E+00 | 1.13E+00 | 1.14E+00 | 8.61E-01 | 8.47E-01 | 1.09E+00 |
|  | 0.5 | 1.39E+00 | 1.49E+00 | 1.44E+00 | 1.40E+00 | 1.07E+00 | 1.08E+00 | 1.31E+00 |
|  | 0.6 | 1.81E+00 | 1.78E+00 | 1.71E+00 | 1.66E+00 | 1.32E+00 | 1.26E+00 | 1.59E+00 |
|  | 0.7 | 2.06E+00 | 2.05E+00 | 1.99E+00 | 2.01E+00 | 1.57E+00 | 1.63E+00 | 1.89E+00 |
|  | 0.8 | 2.37E+00 | 2.42E+00 | 2.31E+00 | 2.35E+00 | 1.79E+00 | 1.68E+00 | 2.15E+00 |
|  | 0.9 | 2.99E+00 | 2.73E+00 | 2.50E+00 | 2.62E+00 | 1.98E+00 | 1.97E+00 | 2.46E+00 |
|  | 1 | 2.96E+00 | 3.24E+00 | 2.70E+00 | 2.92E+00 | 2.43E+00 | 2.14E+00 | 2.73E+00 |

The intra-precision of the method was determined by a sixfold measurement of the peptide mix with concentrations of 0.05, 0.2 and 0.7 µM within one day. Quantification was performed using the calibration curve. The method precision was calculated according to:

$$\text{s}_{\text{Meth}}\text{=}\sqrt{\text{ }\frac{{\sum\text{(x}_{\text{j}}\text{-}{\bar{\text{x}}}_{\text{j}}\text{)}}^{\text{2}}\text{ }}{\text{n-1 }}}$$

 (1)

With x_j_ = values within one day, $\bar{x}_{j}$ **= mean value within one day and n = number of samples.** For inter-precision, samples with concentrations of 0.05, 0.2 and 0.7 µM were prepared six times on each day for three days. The standard deviation within a series (sw), between the series (sb) and the total standard deviation (st) were calculated from the experimental data according to the following equations:

$s_{j}\mathbf{=}\sqrt{\frac{{\sum{(x}_{j}-\bar{x}_{j})}^{2}}{n-1}}$ (2)

$s_{w}=\sqrt{\frac{\sum(s_{j}^{2})}{n}}$ (3)

$s_{b}=\sqrt{\frac{{\sum{(\bar{x}}_{j}-\bar{x}_{\mathrm{ges}})}^{2}}{n-1}}$ (4)

$s_{t}=\sqrt{s_{w}^{2}+s_{b}^{2}}$ (5)

With x_j_ = values within one day, $\bar{x}_{j}$ = mean value within one day and n = number of samples, $\bar{x}_{\mathrm{ges}}$= over all mean value within one day and sj= standard deviation within one day.

**Table S5.** Mean values and standard deviation for determination of the method precision (**tandem mass spectrometer QTRAP^®^6500^+^ of Sciex)**

| **peptide** | **Concentration [µM]** | **Average [µM]** | **s_meth_ [µM]** |
| --- | --- | --- | --- |
| α factor | 0.05 | 0.05 | 0.003 |
|  | 0.2 | 0.203 | 0.008 |
|  | 0.7 | 0.676 | 0.008 |
| CSF | 0.05 | 0.049 | 0.002 |
|  | 0.2 | 0.198 | 0.009 |
|  | 0.7 | 0.712 | 0.008 |
| P-factor | 0.05 | 0.050 | 0.003 |
|  | 0.2 | 0.201 | 0.009 |
|  | 0.7 | 0.676 | 0.013 |

**Table S6.** Mean values and standard deviation for determination of the measurement precision (tandem mass spectrometer QTRAP®6500+ of Sciex)

| peptide | Concentration [µM] | Average [µM] | S_w_ [µM] | S_b_ [µM] | S_t_ [µM] |
| --- | --- | --- | --- | --- | --- |
| α factor | 0.05 | 0.05 | 0.002 | 0.001 | 0.003 |
|  | 0.2 | 0.203 | 0.005 | 0.005 | 0.008 |
|  | 0.7 | 0.676 | 0.014 | 0.002 | 0.008 |
| CSF | 0.05 | 0.049 | 0.002 | 0.001 | 0.002 |
|  | 0.2 | 0.198 | 0.006 | 0.003 | 0.009 |
|  | 0.7 | 0.712 | 0.013 | 0.006 | 0.008 |
| P-factor | 0.05 | 0.050 | 0.002 | 0.001 | 0.003 |
|  | 0.2 | 0.201 | 0.007 | 0.001 | 0.009 |
|  | 0.7 | 0.676 | 0.012 | 0.001 | 0.013 |

**Table S7.** Mean values and standard deviation for determination of the robustness (**tandem mass spectrometer QTRAP^®^6500^+^ of Sciex)**

| **peptide** | **Concentration [µM]** | **Average [µM]** | **s [µM]** |
| --- | --- | --- | --- |
| α factor | 0.05 | **0.056** | **0.001** |
|  | 0.2 | **0.184** | **0.005** |
|  | 0.7 | **0.690** | **0.010** |
| CSF | 0.05 | **0.052** | **0.002** |
|  | 0.2 | **0.169** | **0.005** |
|  | 0.7 | **0.637** | **0.020** |
| P-factor | 0.05 | **0.062** | **0.001** |
|  | 0.2 | **0.169** | **0.002** |
|  | 0.7 | **0.636** | **0.011** |


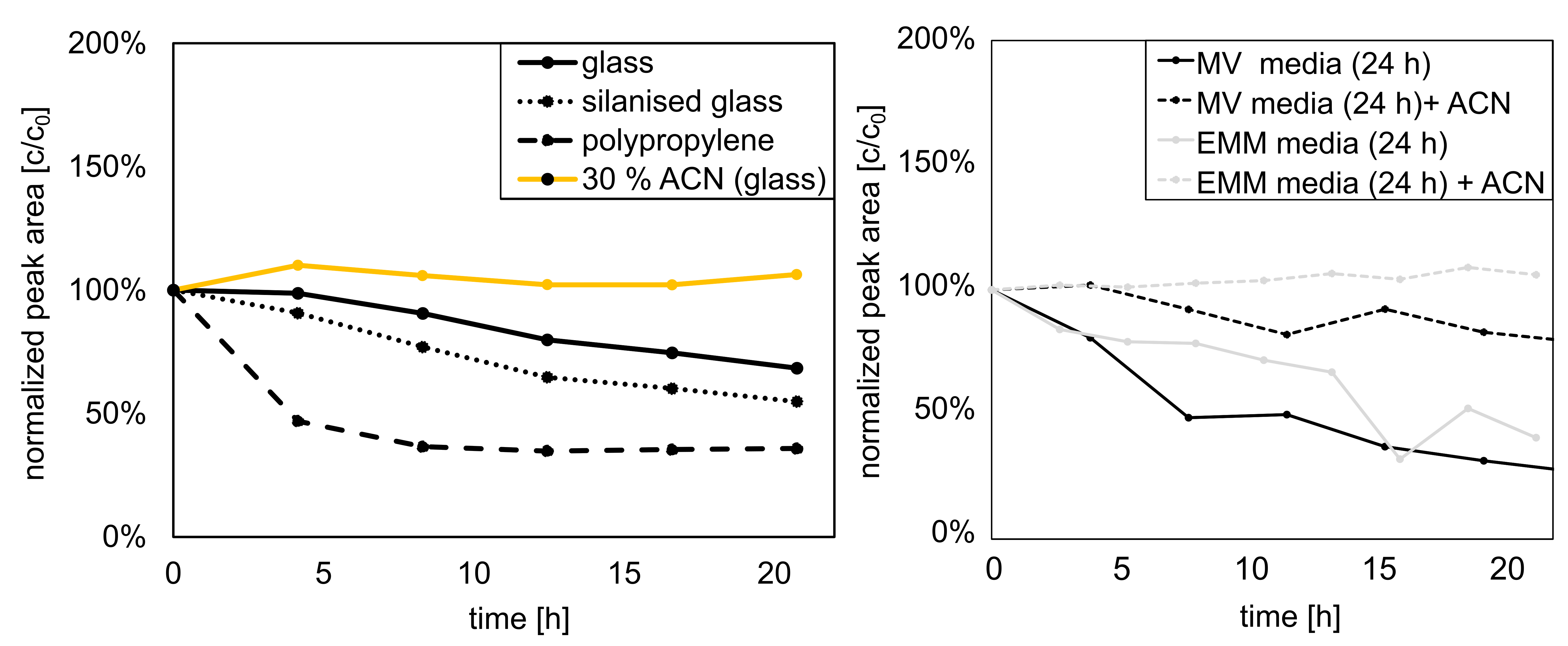


**Fig. S7.** Demonstration of the transferability of the validated method to P-factor. (a) Investigation of sorption tendency to the surface of the vessel (peptides dissolved in LC-MS water + 0.125 % FA) and with the addition of ACN into glass vessels to reduce adsorption as developed. (b) Investigation of peptide stability in EMM and MV media without and with 30 % ACN in MV media and 50 % ACN in EMM media,

**Table S8.** Summary of validation parameters for P-factor (**tandem mass spectrometer QTRAP^®^6500^+^ of Sciex*)***

| Parameter | Peptide | Value | | | | | | |
| --- | --- | --- | --- | --- | --- | --- | --- | --- |
| Linearity (0.01 – 1 µM) | P-factor | y = 2.7139x – 0.0076; R = 0.9995 | | | | | | |
| Presicion (RSD [%]) | Concentration [µM] | 0.05 | | 0.2 | | | 0.7 | |
|  | P-factor | 6.0 | | 4.7 | | | 1.9 | |
| Robustness (0.2 µM, RSD [%]) | matrix | H_2_O | MV | | Yeast MW | W0 | | Yeast W0 |
|  | P-factor | 3.8 | 3.0 | | 0 | 0 | | 1.5 |
| LOD | P-factor | 0.01 µM | | | | | | |
| LOQ | P-factor | 0.02 µM | | | | | | |
